# STING-mediated antiviral response: insights into MVA replication control in avian cells

**DOI:** 10.1128/spectrum.00075-25

**Published:** 2025-09-22

**Authors:** Teresa Brusco, Valentina Menci, Carmen Caiazza, Anna Maria Petrone, Renata Palladino, Matteo Faticanti, Veronica Bignone, Concetta Ambrosino, Elisa Scarselli, Massimo Mallardo, Loredana Siani, Valentino Ruzza

**Affiliations:** 1 Department of Molecular Medicine and Medical Biotechnology, University of Naples Federico II 9307, Naples, Italy; 2 NousCom Srl, Rome, Italy; 3 Department of Biology, University of Naples Federico II 9307, Naples, Italy; Thomas Jefferson University, Philadelphia, Pennsylvania, USA

**Keywords:** viral-host interaction, STING, MVA, avian cells, innate immunity

## Abstract

The safety-tested Modified Vaccinia virus Ankara (MVA) is a well-characterized mutant virus widely used in fundamental research to elucidate the functions of Poxvirus host-interaction factors. Beyond its safety profile, MVA is an attractive viral vector for vaccine development due to its genetic stability and ability to efficiently infect antigen-presenting cells, such as dendritic cells and tumor cells. In this report, we investigated the interplay between MVA and the cyclic GMP-AMP synthase-stimulator of interferon genes (cGAS-STING) antiviral pathway in chicken fibroblast cell lines (wild-type DF-1 and knock-out STING) to verify whether manipulation of the STING axis could impact MVA replication and cell responses. Our findings demonstrate that STING-mediated signaling plays a role in contrasting the replication of MVA. Upon MVA infection, the loss of STING hampered the expression of type I interferons (IFNs) and, in turn, interferon-stimulated gene 15 (ISG15) and interferon-induced transmembrane protein 3 (IFITM3). In line with these results, the expression of early and late MVA genes was enhanced, and DNA replication occurred earlier and was more abundant. Interferon regulatory factor 1 (IRF1) and myeloid differentiation primary response 88 (MyD88) were significantly induced by MVA infection in STING-KO cells, indicating that their responses to MVA infection are independent of the cGAS/STING axis. Collectively, these results refine our knowledge of MVA-host interaction in chicken fibroblasts and offer insights to guide strategies for enhancing Poxvirus vaccine vector production.

**IMPORTANCE:**

Given the context-dependent nature of STING antiviral activity, it is critical to broaden the investigation in order to clarify the virus-host response mechanisms across different species, particularly in chicken fibroblasts, to provide insights into MVA-based vaccine production improvements.

## INTRODUCTION

The properties that made Modified Vaccinia Ankara (MVA) virus an attractive antigen-delivery vehicle are essentially attributable to the high-level transgene expression and the cargo capacity (1), the avirulence and inability to replicate in human cells (2), and the general safety profile, already observed in the over 120,000 healthy volunteers vaccinated during the last smallpox eradication campaign (1, 3).

MVA was derived in the seventies from the parental strain Chorioallantois Vaccinia Ankara (CVA) virus after more than 570 sequential passages in chicken embryo fibroblast cells (CEFs) (4, 5). During this adaptation, MVA lost approximately 30 Kbp of its genome, consisting of six major deletions and further gene truncations/mutations. These changes were shown to be decisive for its attenuated virulence, restricted host range replication, and safety while preserving its high immunogenicity as a viral vector (6, 7).

Although Vaccinia virus has the ability to contrast host innate immune response by releasing soluble inhibitors that interfere with cytokine, chemokine, and interferon activities (8–10), MVA has the unique immunological property to stimulate adequately host innate immune response without releasing any interferon α/β (IFNα/β), interferon γ (IFNγ), and tumor necrosis factor (TNF) inhibitor protein, thus representing an interesting profile for an immunogenic but safe vaccine (8).

Indeed, MVA stimulates IFNα/β production in murine conventional dendritic cells mostly via the cytosolic DNA-sensing axis cyclic GMP-AMP synthase/ stimulator of interferon genes (cGAS)/STING/Interferon regulatory factor 3 (IRF3), with interferon regulatory factor 7 (IRF7) and interferon alpha and beta receptor subunit 1 (IFNAR1) amplifying the signal, but with the toll-like receptor 7−9/myeloid differentiation primary response 88 (TLR7-9/MyD88) pathway playing a minor role in activating innate immune response (9).

The STING is a transmembrane protein located in the endoplasmic reticulum and encoded by the transmembrane protein 173 (TMEM173) gene (10). Initially identified as a crucial player in host defense against viral infections (11), it has since been implicated in a broader range of innate immune processes, as well as in adaptive immunity regulation and in immunotherapy (12, 13). Cytosolic DNA, originating either from infections, endogenously through phagocytosis, or released from mitochondria, initiates the production of 2′3′-cGAMP (cG[2′–5′]pA[3′–5′]p) via the enzyme cGAS (14). This 2′3′-cGAMP is recognized by the adaptor protein STING, facilitating its translocation to the Golgi apparatus and promoting the interaction with TANK-binding kinase 1 (TBK1), which next activates the transcription factor IRF3 (15, 16). IRF3 dimerizes and enters the nucleus to initiate a type I Interferon (IFN-I) response, leading to the expression of a set of interferon-stimulated genes (ISGs) and the establishment of antimicrobial immunity and inflammatory state (17). Moreover, the activation of cGAS-STING signaling also leads to nuclear factor kappa-light-chain-enhancer of activated B cells (NF-κB) activation, whose regulation relies upon TNF receptor-associated factor 6 (TRAF6), NF-κB essential modulator (NEMO), IkappaB kinase beta (IKKβ), and TBK1 (18). Thus, the immune pathway generated by the dsDNA-sensing cGAS-STING can both counteract viral replication and spreading. As confirmation of these issues, we have previously demonstrated that STING knockout (KO) cell lines are more permissive to the infection of an oncolytic herpes simplex virus (HSV) compared with their wild-type counterparts (19). Moreover, in the STING knockout cell line, the HSV spread was faster and evidenced the formation of larger lysis plaques compared with the wild-type cell line (19).

In this article, we demonstrate that STING-mediated signaling plays a role in contrasting the replication of MVA in the spontaneously immortalized chicken embryo fibroblast DF-1 cells. Remarkably, following MVA infection in **D**F-1 **S**TING **K**nock-out (DSK) cells, we observed reduced expression of IFNα and IFNβ genes along with Interferon-stimulated gene 15 (ISG15), a major protein induced by type I interferons (20), interferon-induced transmembrane protein 3 (IFITM3), which interferes with vaccinia virus entry into the cytoplasm (21), and other interferon-stimulated genes (ISGs).

Consequently, upon MVA infection, DSK cells exhibit more cytoplasmic replication centers, higher expression of early and late viral genes, improved transgene expression, and an overall increase in viral titer compared with the DF-1 WT cells.

Notably, TLR signaling genes interferon regulatory factor 1 (IRF1) and MyD88 are significantly induced by MVA infection in DSK cells, probably to counterbalance the reduced IFN-I response of the cytosolic DNA-sensing cGAS/STING/IRF3 axis.

## RESULTS

### STING knockout cell line generation and characterization

**Fig 1:**
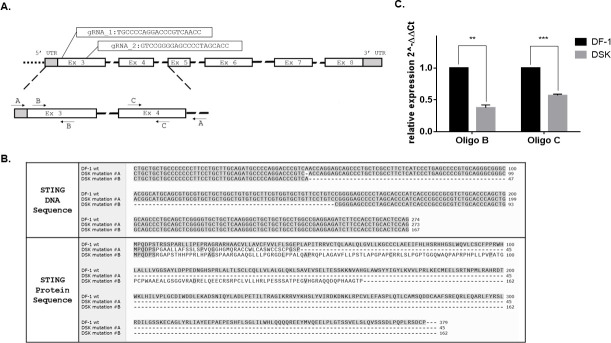
DF-1 STING KO cell line generation. (**A**) Schematic representation of the chSTING locus and the gRNA’s position. A portion of the gene is zoomed in and shows oligo pairs positions: oligo pair A was used for genomic sequencing of the locus during clone screening; oligo pairs B and C were used for STING gene expression analysis. (**B**) DNA and protein sequence alignments between wild-type DF-1 and the heterozygous mutant alleles of DSK cells. (**C**) Expression analysis of STING by qRT-PCR in DF-1 WT (black) and DSK (gray). STING levels are relative to GAPDH. Data are shown as mean ± SEM (*N* = 2). **P  <  0.01, ***P  <  0.005

To investigate the cGAS-STING signaling role as mediator of the cellular response upon MVA infection, a DF-1 chSTING KO cell line was generated. Two guide RNAs (gRNAs) targeting the third exon of the STING chicken gene were designed (Fig. 1A), and each gRNA was cloned into the PX458 plasmid. The resulting plasmids contain the gRNA and the Cas9 protein fused at the N-terminus with an enhanced green fluorescent protein (EGFP) tag. Following co-transfection with the two plasmids, single-cell clones were isolated by limiting dilution and screened by PCR (Fig. S1) using primers designed to amplify a region spanning from the 5’ untranslated region (UTR) to the intron 4 of the genomic DNA sequence (Fig. 1A, zoomed-in section). One clone, designated DSK, exhibited heterozygous mutations in the STING locus. As confirmed by Sanger sequencing, the mutations in this clone consist of a single point deletion on one allele and a 107 bp deletion on the other, thus resulting in predicted nonfunctional peptides, lacking all the functional domains (i.e., the serine 366) and even the first transmembrane and loop domains essential for the ER translocation (Fig. 1B). Consequently, quantitative PCR (qPCR) analysis revealed reduced STING transcriptional levels (Fig. 1C), using both oligo pairs within the 107 bp deletion sequence and targeting exon 4 (Fig. 1A). Taken together, these results indicate that in DSK cells, the STING function is impaired.

### Disruption of the STING gene enhances MVA viral growth

**Fig 2:**
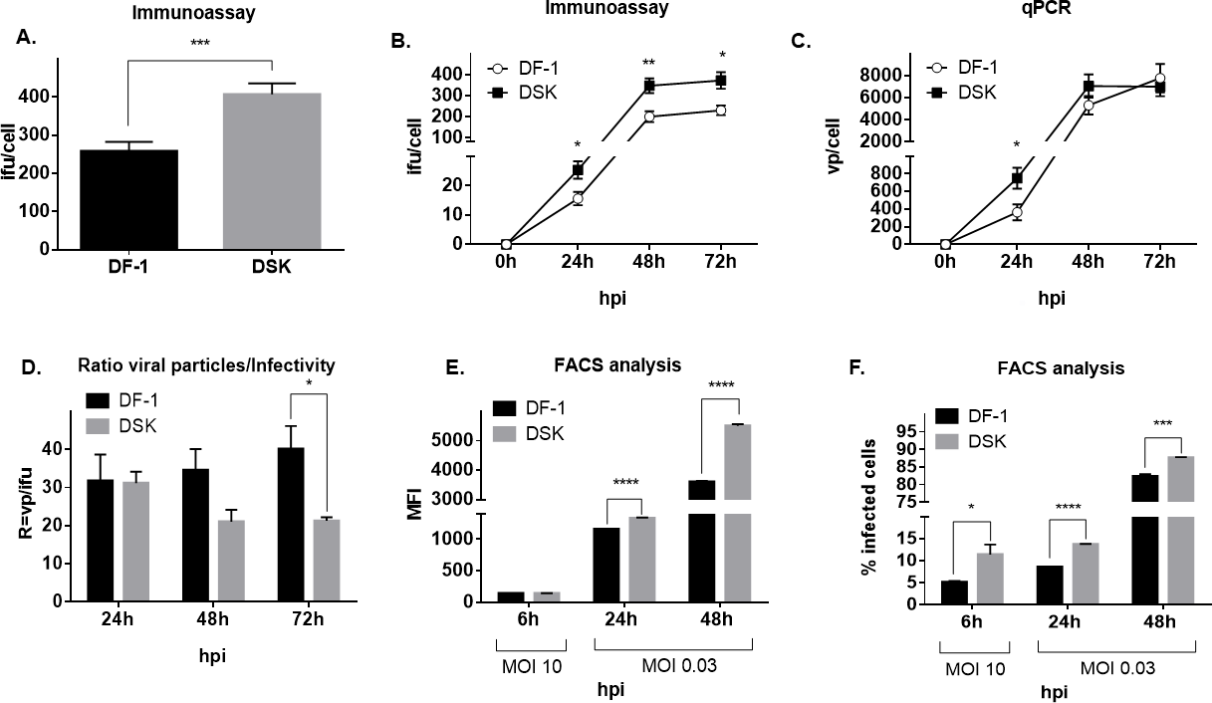
Virus entry and replication assay. (**A**) MVA-red infectious titer at 72 h post-infection in DF-1 WT and DSK cells. (**B and C**) MVA-red titration by immunoassay and qPCR during time course infection in DF-1 WT and DSK cells. Hours post-infection are reported on the x-axis. (**D**) MVA-red ratio vp/ifu during time course infection in DF-1 WT and DSK cells. Hours post-infection are reported on the x-axis. Percentage of infected cells (**E**) and MFI (**F**) analyzed by flow cytometry at 6 (cells infected at MOI 10), 24, and 48 h post-infection (cells infected at MOI 0.03) in DF-1 WT and DSK cells. Hours post-infection are reported on the x-axis. Broken axis histograms allow us to appreciate low values. Data are shown as mean ± SEM (*N* ≥ 3). *P  <  0.05, **P  <  0.01, ***P  < 0.001, ****P < 0.0001.

In order to evaluate whether the STING knock-out had an impact on MVA viral cycle, a viral growth assessment by infecting DSK and DF-1 cells at MOI (Multiplicity Of Infection) 0.03 with MVA expressing HcRed fluorescent protein (MVA-red) was performed. Seventy-two hours post-infection, the cells were collected by scraping and lysed with one freeze-thaw cycle and sonicated for 10 minutes before the virus was titrated by immunoassay (Fig. 2A). The analysis revealed a 1.6-fold increase in the infectious unit per cell (ifu/cell) production in DSK compared with DF-1 WT cells. To better characterize the viral growth increase, an infection time-course experiment was performed, harvesting cells at 24, 48, and 72 h post-infection. Immunoassay titration confirmed an approximately 1.6-fold increase in MVA ifu/cell titer in DSK not only at 72 h post-infection but also at 24 and 48 h post-infection (Fig. 2B). However, since the infectious titer may not correlate with viral DNA levels, a qPCR assay amplifying the MVA DNA polymerase gene (E9L) was performed to measure only encapsidated viral genomes. The results showed that the viral particle per cell (vp/cell) concentration was similar between KO and WT cells at 48 and 72 h, whereas at 24 h, it was approximately 1.9 times higher in DSK compared with WT cells (Fig. 2C). Considering the flattening of the viral particle concentration at 48 and 72 h, the virus exhibits higher infectivity in DSK cells at later time points as indicated by the vp/ifu ratio (Fig. 2D).

To support these observations, the cells were infected with MVA-red at different MOIs and harvested at 6, 24, and 48 h post-infection for flow cytometry analysis, looking at the expression of the viral red fluorescent protein. As shown in Fig. 2E, mean fluorescent intensity (MFI) measurements revealed no differences at 6 h but were significantly higher in DSK compared with DF-1 WT cells at later time points. On the other side, Fig. 2F revealed that DSK cells exhibited a higher number of infected cells compared with DF-1 WT at all three times of harvest.

Together, these findings demonstrate that the absence of STING promotes MVA viral growth and infectivity. Furthermore, the higher percentage of infected cells at 6 h may reflect the enhanced expression of early genes in STING-lacking cells.

### Early and late MVA gene expression is enhanced in DSK-infected cells

**Fig 3:**
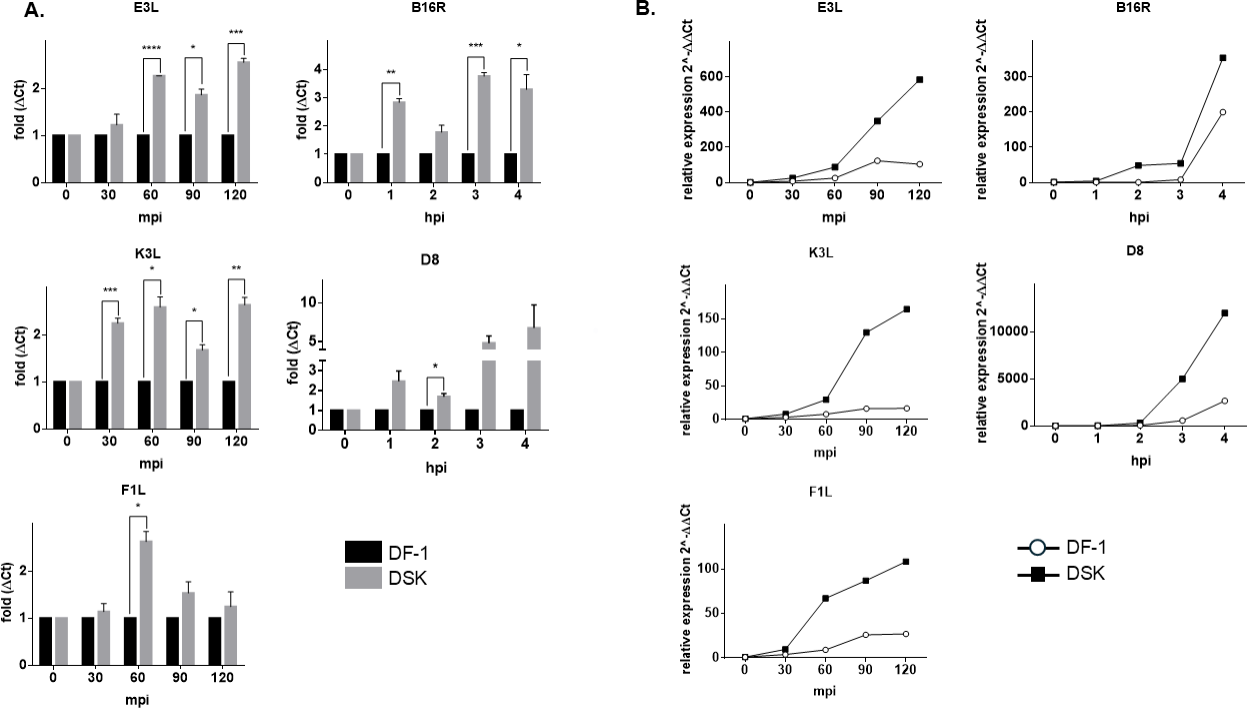
MVA early and late gene expressions in DF1- WT and DSK cells. DF-1 and DSK cells were incubated with MVA at 4°C for 30 min, washed with cold phosphate-buffered saline (PBS), and cultured with the growth medium at 37°C for the indicated times before harvesting for RNA collection. Relative quantity of MVA early (E3L, K3L, and F1L) and late gene (B16R, D8) was measured by qRT-PCR (**A and B**). In panel A, data from three independent experiments are shown as means with relative expression levels normalized to DF-1 cells for each time point to allow comparison between the cell lines. In panel B, a representative experiment out of three independent replicates is shown with normalization to time 0 for each cell line to highlight expression kinetics over time. *P < 0.05, **P < 0.01, ***P < 0.005 ****P < 0.0005).

In order to assess whether *STING* loss of function affects early and late stages of the MVA replicative cycle, the expression of early and late viral genes was analyzed in infected DF-1 WT and DSK cells. We selected E3L, K1L, and F1L as MVA early genes, and B16R and B8 as late genes. To avoid interference in gene expression analysis due to the presence of multiple viral particles in a single cell, DF-1 and DSK cells were infected at 0.2 MOI. Cells were harvested at different time points, and the total RNA was analyzed by quantitative reverse-transcription PCR (qRT-PCR). As shown in Fig. 3 (panels A, B), all the tested early genes showed earlier and more abundant expression in DSK cells compared with the WT. This effect was evident as early as 30 min post-infection, reaching peak fold induction at 60 min, whereas at 2 h post-infection, the differences in the early gene expression between DF-1 and DSK were observed to a lesser extent. Similarly, the late gene expression showed an increase in the expression of the DSK cells from 2 to 4 h post-infection, peaking at 3 h (B16R) and 4 h (D8) (Fig. 3A and B).

### The lack of STING influences MVA DNA replication

**Fig 4:**
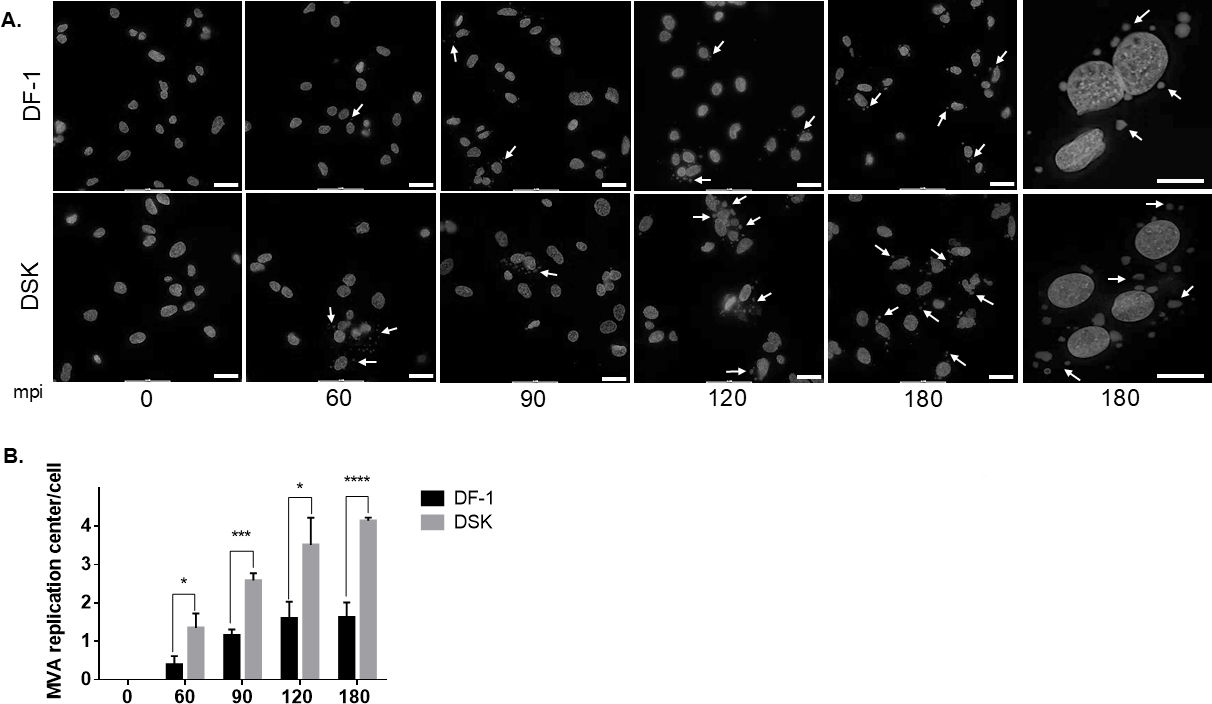
The effect of STING lacking on viral replication of MVA. (**A**) DF-1 and DSK cells were incubated with 0.1 MOI of MVA for 60, 90, 120, and 180 min at 37°C, followed by Hoechst staining. The dots around the nuclei indicated by arrows represent the MVA replication centers. Scale bars 20 µm. (**B**) Quantitative analysis of replication centers was conducted by normalizing to the cell count. *P < 0.05, ***P < 0.005, ****P < 0.0005.

We have previously demonstrated that there is a strict correlation between Vaccinia Virus early genes expression and the replication of viral DNA (22). Thus, we wanted to assess whether the lack of STING could also affect the DNA replication of MVA. To achieve this, MVA infection with 0.1 MOI was performed, harvesting the cells at the indicated time points post-infection. Viral entry was synchronized as described in the methods, and following incubation for 15 min at 37°C, the unbound viral particles were washed. As shown in Fig. 4A, in DSK cells, some DNA replication centers were already visible at 60 min post-infection (mpi) compared with the 90–120 mpi observed in DF-1 cells. Moreover, the number of DNA replication centers per nucleus was higher and larger in size in the DSK cells at 90 mpi, reaching peak induction at 120 mpi (Fig. 4B).

Altogether, these data confirm that STING is also involved in the modulation of MVA replication center number and formation.

### Analysis of STING downstream gene expression upon MVA infection

**Fig 5:**
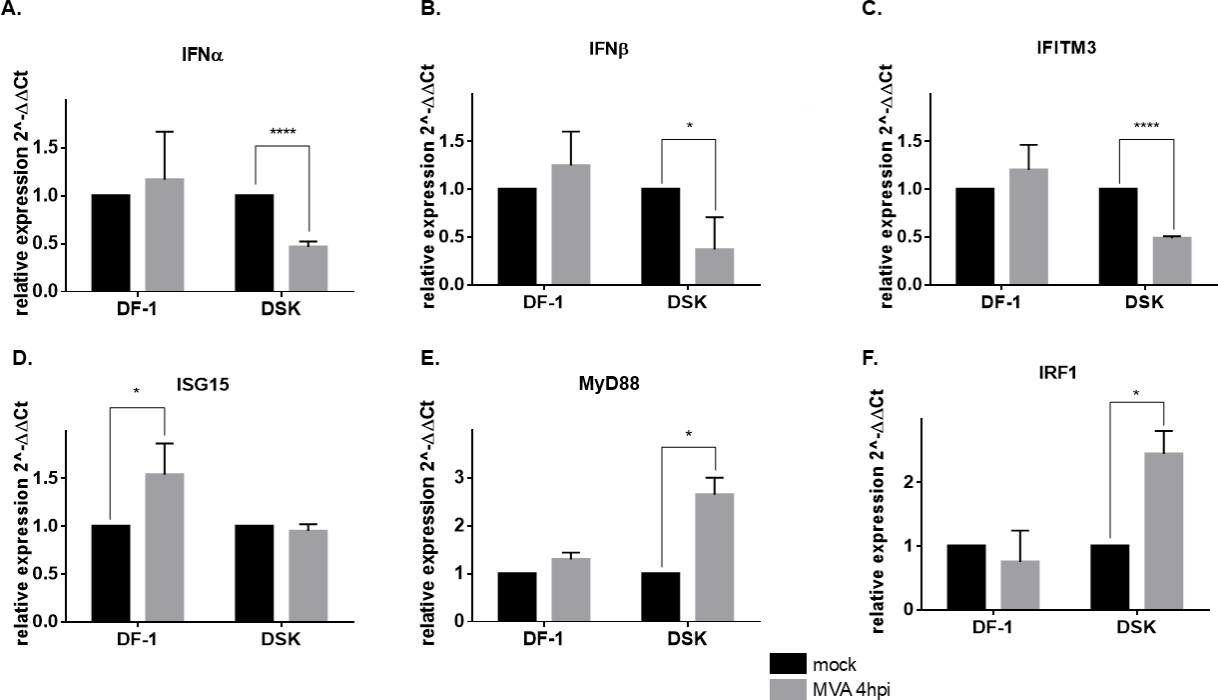
STING downstream gene expression upon MVA infection. Quantitative analysis of gene expression involved in the response to MVA infection in DF1 and DSK cells. Error bars reveal the standard deviation from three biological repeats. *P < 0.05; ****P < 0.0001 compared with the level of mock non-treated cells.

Several genes are regulated by STING activation upon viral infection. Among them, we wanted to analyze the expression of interferon α and β, IFITM3, ISG15, MyD88, and IRF1 in DF-1 and DSK cells upon MVA infection and verify whether their expression is affected when STING functionality is impaired. Type I interferon subtypes IFNα and IFNβ are transcriptionally activated by the infection-mediated phosphorylation of STING (23, 24). By qRT-PCR, we observed that both genes are activated at 4 h post-MVA infection in DF-1 cells. Conversely, in DSK cells, the INFα and INFβ expression was downregulated at 4 h post-MVA infection (Fig. 5A and B). The same result was observed when analyzing the expression of IFITM3 (Fig. 5C), a gene that is induced by STING via the activation of type I IFN (25). We further analyzed the expression of ISG15, a gene encoding for a ubiquitin-like molecule that is highly induced by type I IFN during infection by viral and bacterial pathogens (26). As expected, ISG15 was upregulated by MVA infection in DF-1 cells, whereas its expression rate was comparable in mock-infected vs. infected DSK cells (Fig. 5D). The myeloid differentiation marker MyD88 is able to complex with STING, preventing its autophagic degradation (27). Moreover, MyD88-STING complex is required for lipopolysaccharide (LPS)-induced aconitate decarboxylase 1 (ACOD1) expression during septic shock. We wanted to investigate the expression of MyD88 upon MVA infection. As shown in Fig. 5E, MyD88 is slightly upregulated at 4 h post-infection in the DF-1 cells, whereas it is strongly upregulated in the DSK cells. Finally, we analyzed the expression rate of IRF1. Our analysis reveals that at 4 h post-MVA infection, IRF1 expression is specifically upregulated in DSK cells, whereas no such upregulation is observed in WT DF-1 cells (Fig. 5F). These findings collectively demonstrate that in chicken fibroblasts, impairment of STING function significantly affects the primary downstream effectors of the cGAS/STING signaling pathway.

## DISCUSSION

MVA has the unique immunological property to fine-stimulate host innate immune response, representing an interesting profile for an immunogenic but safe vaccine (9).

STING is one of the key host genes, mediating the innate immune response upon MVA infection (9). However, the mechanisms behind its role in counteracting and restricting viral replication remain not completely understood.

Specifically, the role of the cGAS-STING signaling pathway in activating antiviral responses within chicken cells following MVA infection has yet to be thoroughly investigated (28). Indeed, we generated a **D**F-1 **S**TING **K**nock-out (DSK) cell line by means of CRISPR CAS9-mediated genome editing technique and evaluated the impact on MVA replication efficiency and cell interferon response in comparison to wild-type counterpart.

The loss of STING activity in DSK cells leads to a significant increase in the infective titer of MVA across all tested time points (Fig. 2B). Conversely, the number of genome particles per cell in DSK cells is higher only during the initial phase of infection; over time, it becomes comparable with that of wild-type cells, suggesting that the DNA replication curve has likely reached a plateau (Fig. 2C). As a result, during the initial phase of infection, the ratio of viral particles to infectious particles (vp/ifu) is similar between DSK and DF-1 cells; however, at later stages, this ratio is significantly lower in DSK cells, indicating higher infectivity (Fig. 2D). These data suggest that STING may have a dual impact on the MVA replication cycle, affecting both the initial phases of infection and the processes of viral assembly and maturation at the ER and/or trans-Golgi network levels.

The ability of MVA to replicate in the absence of STING was also evaluated through flow cytometry, by infecting DSK and DF-1 cells with an MVA-red virus at both high (10) and low (0.03) MOI, examining both the early and late phases of infection.

At high MOI and during the early phase of infection, the percentage of fluorescent cells is meaningfully higher in DSK cells, indicating that MVA replication is enhanced in a STING knockout (KO) cell background (Fig. 2F). However, the MFI at this time point is comparable between the two cell lines (Fig. 2E), probably because the selected time point is still too premature to detect a measurable increase in mean fluorescence intensity between WT and DSK cells. At low MOI and late infection phase, an increase in both fluorescent cell percentage and MFI in DSK compared with DF-1 cells can be appreciated, highlighting again the inhibiting impact of STING on MVA replication and maturation.

In summary, with high MOI at the early phase of infection, it is evidenced that MVA gene expression is faster in the absence of STING activity. On the other hand, in the advanced phase of infection, both the percentage of the infected cells and the MFI are consistently higher in DSK cells, highlighting that STING can intervene at different steps of the MVA replication cycle.

A distinctive feature of Poxviruses lies in their ability to replicate in the cytoplasm of the host cell. In particular, vaccinia virus localizes in discrete cytoplasmic nuclei called virosomes or factories (29), where, after DNA replication and late gene expression (~6 h), the intracellular mature virus (IMV) assembly starts with the support of membranes borrowed from the ER. Later on, some extracellular enveloped virus particles are eventually formed in the trans-Golgi network (29).

We observed a notable increase in the number of MVA factories in DSK compared with DF-1 cells, detectable as early as 60 min post-infection (mpi). By 180 mpi, DSK cells show an average of ~3–4 factories per cell, whereas DF-1 cells exhibit less than one (Fig. 4A and B). These findings further support the hypothesis that MVA replication is enhanced in a cellular environment lacking STING activity.

MVA early gene transcription begins within 20 min of infection, and approximately 2 h later, DNA replication and the transcription of late genes start. By 6 h, new virus particles are ready to be assembled (29). To further investigate the dynamics of STING’s impact on MVA replication, we assessed the kinetics of early and late viral gene expressions in DSK compared with DF-1 cells (Fig. 3A and B). The results showed that in DSK cells, both early and late gene expressions were higher, indicating that the absence of STING enhances the early stages of the viral replication cycle. However, the expression of early, intermediate, and late genes is not synchronous because some genes are expressed at multiple stages due to their hybrid promoters containing more than one promoter motif (early and late), which have not been completely characterized yet (30).

Microarray analyses revealed the induction of host resistance and immune modulation genes following infection of HeLa cells with MVA (31). To further explore the mechanisms of MVA infection, we assessed the interferon response and interferon-stimulated genes, including interferon α and β, IFITM3, ISG15, MyD88, and IRF1 in DSK cells and examined how their expression is affected when STING functionality is impaired.

Type I interferons α and β are the main effector genes of the antiviral c-GAS-STING pathway (24). Using qRT-PCR, we analyzed their expression levels after 4 h of MVA infection in the DF-1 and DSK cell lines. As expected, both genes were upregulated in the WT DF-1, whereas the ablation of STING impaired their expression (Fig. 5A and B). Interferon-inducible transmembrane 3 (IFITM3) is an intrinsic antiviral effector able to restrict viral cytosolic entry by blocking endosomal and membrane fusion pathways. Indeed, IFITM3 overexpression significantly reduces vaccinia virus (VACV) levels in 293T and Vero cells (21, 32). Here, we demonstrate that upon MVA infection, mRNA levels of IFITM3 are reduced in DSK cells compared with DF-1 (Fig. 5C), highlighting the role of STING in amplifying and sustaining antiviral response following Poxvirus infection. Moreover, the MVA-mediated upregulation of the interferon-stimulated gene 15 (ISG15) observed in the DF-1 was also hampered in the DSK cells. The lack of ISG15 activation can be explained by the fact that it is highly induced by type I IFN. It has been shown that in ISG15-deficient THP-1 cells, the infection of HIV-1 was enhanced in both undifferentiated and phorbol-12-myristate-13-acetate (PMA)-differentiated ISG15-deficient THP-1 cells compared with the control (33). Thus, the lack of STING, affecting the expression of type I IFN and ISG15, may explain the observed enhancement of MVA replication.

MyD88 and STING may form a complex that acts as adaptor proteins in innate immunity (27). However, it has been shown that the depletion of STING had no effect on the level of MyD88 protein. As we observed that MyD88 is only slightly upregulated at 4 h post-infection in the DF-1 cell but is strongly upregulated in the DSK cells, we may speculate that the mechanism by which MyD88 is involved in the innate immunity is not mediated by the transcriptional regulation of the encoding gene. Moreover, we analyzed the expression rate of the interferon regulatory factor 1 (IRF-1). Surprisingly, we found that IRF1 is not transcriptionally activated in DF-1, whereas its transcription is enhanced in DSK cells. However, it is reported that MVA infection induces the production of IFN-I in dendritic cells, mainly via STING activation of the transcription factors IRF3 and IRF7 (27). In addition, it is reported that TLR9-triggered MyD88 activation caused not only nuclear translocation of IRF1 but also activated its transcription factor function in murine macrophages (34). In summary, in chicken cells, we observed that IFN-I-mediated activation of MyD88/IRF1 occurs only in the absence of STING, highlighting that this is secondary to the IRF3/IRF7 pathway in responding to MVA infection.

### Conclusions

Overall, several pieces of evidence suggest that STING plays multiple roles in counteracting MVA during the viral replication cycle.

First, viral replication is facilitated in DSK cells, as evidenced by a higher number of infective and viral particles during the early phase of infection, an early and substantial increase in both early and late viral gene expression, and a concurrent increase in the number of cytosolic replication centers.

Second, the initial augmented number of viral particles in DSK cells tends to equalize the number of viral particles in DF-1 cells, probably due to a saturation phenomenon. Nevertheless, the DSK cells present an advantage during the viral maturation phase as the number of infective particles is significantly and constantly higher than that of DF-1, indicating that STING intervenes not only at the viral entry phase but also during the final morphogenesis of new infectious virions.

Interestingly, it has been demonstrated that STING does not impact the replication of MVA in the permissive cell line BHK21. In particular, the growth kinetics of MVA in BHK21 *STING KO* cells are indistinguishable from those shown by their wild-type counterpart (35). In BHK21 cells, although the expression of some ISGs is upregulated upon MVA infection, the cGAS/STING axis does not serve as the primary mediator of the antiviral response, suggesting alternative sensing mechanisms.

Conversely, in our report, we show that the transcriptional activation of type I IFN, IFITM3, and ISG15 is hampered in MVA-infected DSK cells. Moreover, it has been shown that in duck cGAS-KO fibroblasts, duck adenovirus replication is enhanced, accompanied by the inactivation of type I IFN and ISGs, suggesting a species-specific response downstream the cGAS/STING axis (36).

Given the context-dependent nature of their antiviral activity, it is critical to broaden the investigation in order to clarify the virus-host response mechanisms in the different species, particularly in chicken fibroblasts, to give insights into MVA-based vaccine production improvements.

In summary, the results suggest that STING could counteract MVA at different levels of its replication cycle. Interestingly, by using an optimized version of the proximity-dependent biotin identification (BioID) technique applied to detect protein-protein interactions in living cells, Motani and Kosako identified several STING interactors, including IFITM3 (37). Likely, in collaboration with IFITM3, which is dysregulated upon infection in DSK cells, STING may counteract MVA, particularly during the entry phase, by targeting viral particles for elimination through the endosomal pathway.

## MATERIALS AND METHODS

### Cells and viruses

DF-1 cells (spontaneously immortalized chicken embryo fibroblasts) were purchased from ATCC (Collection number: CRL-12203). DF-1 and DF-1 STING−/− cells were cultured in complete Dulbecco’s modified Eagle medium (high glucose, [+] L-glutamine, [+] pyruvate), supplemented with 10% heat-inactivated fetal bovine serum (FBS) and maintained at 37°C in 5% CO_2_.

The cell lines were tested for the absence of mycoplasma contamination by PCR.

Vero cells used for virus titration were cultured in complete Dulbecco’s modified Eagle medium (low glucose, [+] L-glutamine, [+] pyruvate), supplemented with 5% FBS and maintained at 37°C in 5% CO_2_.

MVA expressing HcRed fluorescent protein (MVA-red) was amplified in DF-1 cells and titrated in Vero cells. MVA HcRed virus is described in (38).

MVA-red was sonicated for 10 min prior to infection and titration and used within 30 min.

At harvest, the cells were scraped, and to release the virus, one freeze/thaw cycle was performed, followed by 10 min sonication.

### Plasmid construction and cell transfection

The plasmids used for knocking out STING were obtained as described before (12). Briefly, guide RNAs were cloned into pSpCas9(BB)−2A-GFP (PX458) (Addgene #48138) following BbsI digestion. The following primers were used to generate gRNAs:

STING gRNA1 FW: 5′-CACCGTGCCCCAGGACCCGTCAACC-3′;

STING gRNA 1 REV: 5′-AAACGGTTGACGGGTCCTGGGGCAC-3′;

STING gRNA 2 FW: 5′-CACCGGTGCTAGGGGCTCCCCGGAC-3′;

STING gRNA 2 REV: 5′-AAACGTCCGGGGAGCCCCTAGCACC-3′;

STING gRNA 3 FW: 5′-CACCGTGTCGGCGGCTCAGCCTACC-3′;

STING gRNA 3 REV: 5′-AAACGGTAGGCTGAGCCGCCGACAC-3′.

Double-stranded DNA fragments were produced by mixing 10 µM forward primer, 10 µM reverse primer, 50 mM Tris-HCl, 10 mM MgCl₂, 10 mM dithiothreitol, and 1 mM ATP. The mixture was incubated at 37°C for 30 min, then heated to 95°C for 5 min, and subsequently cooled slowly to room temperature. Double-stranded gRNA was digested with BbsI and cloned into PX458.

DF-1 cells were transfected using Lipofectamine 2000 (Invitrogen), and transfection efficiency was evaluated by looking at GFP expression with a fluorescence microscope. Cell cloning was performed by limiting dilution.

### Genomic DNA isolation and analysis

To perform STING mutation screening of transfected cells, genomic DNA from cell clones and DF-1 WT cells was extracted using DNeasy Blood & Tissue Kit (Qiagen), following the manufacturer’s instructions. PCR was performed using Phusion Hot Start II DNA Polymerase (Thermo Fisher), following the manufacturer’s instructions, with the following primers to amplify the STING genomic region targeted by sgRNA guides: forward 5′-AGCATCCAGAGGAAGTGGAG-3′ and reverse 5′-GCTAAACATCACTGCTGAGTATCC-3′.

PCR products were run on agarose gel, amplicons were excised, and DNA was purified using Wizard SV Gel and PCR Clean‐Up System (Promega), following the manufacturer’s instructions. The DNA sequence was analyzed by Sanger sequencing.

DNA sequences from DF-1-transfected clones were aligned with DF-1 wt sequence.

### Virus titration by qPCR and immunoassay

MVA genome titer was assessed by quantitative PCR on viral DNA polymerase (E9L) using the following primers: forward 5′-CGGCTAAGAGTTGCACATCCA-3′, reverse 5′-CTCTGCTCCATTTAGTACCGATTCT-3′, and probe 5′-AGGACGTAGAATGATCTTGTA-3′ (FAM-TAMRA) (from Baker and Ward, 2014). The qPCR assay was performed using TaqMan Universal PCR Master Mix (Applied Biosystems). A standard curve was generated with a plasmid containing 1,000 bp of the E9L viral gene.

MVA infectious titer was obtained with an immunostaining assay executed on monolayers of Vero cells. At 48 h post-infection, the infected cells were detected using a rabbit polyclonal anti-vaccinia primary antibody (Abcam), followed by an anti-rabbit horseradish peroxidase (HRP)-conjugated secondary antibody (Sigma).

### FACS analysis

For fluorescent-activated cell sorter (FACS) analysis, the cells (infected or not infected) were scraped and centrifuged. After washing cell pellets with PBS, the samples were stained with LIVE/DEAD Fixable Near-IR Dead Cell Stain Kit (Thermo Fisher Scientific) 1:100 in PBS for 15–20’. The cells were centrifuged again and resuspended in PBS. Flow cytometry was performed using BD FACS Canto II, and data were analyzed using FlowJo v10 software.

### Immunofluorescence assay

To assess the micronuclei formation, DF-1 cells were infected with MVA-red at an MOI of 0.1 under the following conditions. The cells were seeded onto glass coverslips and incubated overnight under standard growth conditions. The next day, MVA-red at a MOI of 0.1 was added to the culture medium, and the cells were incubated on ice for 30 min, followed by incubation at 37°C for the designated times. The cells were then washed three times with PBS and fixed with 3.7% formaldehyde for 30 min at room temperature, followed by a 10 min incubation with 0.1 M glycine. Nuclear staining was performed using Hoechst (Invitrogen H3570) for 5 min at room temperature, protected from light. Coverslips were subsequently washed three times with PBS and mounted onto glass slides with a 1:1 PBS/glycerol solution. Images were acquired using a laser scanning microscope (LEICA DMi8) and analyzed using LEICA LAS X software.

### Gene expression analysis

RNA was extracted using Direct-zol RNA Miniprep (Zymo Research), and cDNA synthesis was performed with SuperScript IV VILO Master Mix (Invitrogen) according to the manufacturer’s instructions.

RT-PCR was performed using the PowerUp SYBR Green Master Mix (Applied Biosystems) using the oligonucleotides listed in Table S1.

Relative gene expression was analyzed with the 2^-ΔΔCt^ method using GAPDH as an endogenous housekeeping gene.

### Statistical analyses

Statistical analyses were performed using unpaired *t*-test method. *P* values less than 0.05 were considered significant. The results are represented as mean ± standard error of the mean (SEM).

## SUPPLEMENTAL MATERIAL

Supplemental material spectrum.00075-25-s0001.docx Fig. S1 and S2; Table S1.

ASM does not own the copyrights to Supplemental Material that may be linked to, or accessed through, an article. The authors have granted ASM a non-exclusive, world-wide license to publish the Supplemental Material files. Please contact the corresponding author directly for reuse.

## References

[1] Sutter G, Moss B. 1992. Nonreplicating vaccinia vector efficiently expresses recombinant genes. Proc Natl Acad Sci USA 89:10847–10851. doi:10.1073/pnas.89.22.10847 1438287 PMC50439

[2] Sancho MC, Schleich S, Griffiths G, Krijnse-Locker J. 2002. The block in assembly of modified vaccinia virus Ankara in HeLa cells reveals new insights into vaccinia virus morphogenesis. J Virol 76:8318–8334. doi:10.1128/jvi.76.16.8318-8334.2002 12134037 PMC155139

[3] Stickl H, Hochstein-Mintzel V, Mayr A, Huber HC, Schäfer H, Holzner A. 1974. [MVA vaccination against smallpox: clinical tests with an attenuated live vaccinia virus strain (MVA) (author’s transl)]. Dtsch Med Wochenschr 99:2386–2392. doi:10.1055/s-0028-1108143 4426258

[4] Volz A, Sutter G. 2017. Modified vaccinia virus ankara: history, value in basic research, and current perspectives for vaccine development. Adv Virus Res 97:187–243. doi:10.1016/bs.aivir.2016.07.001 28057259 PMC7112317

[5] Meisinger-Henschel C, Schmidt M, Lukassen S, Linke B, Krause L, Konietzny S, Goesmann A, Howley P, Chaplin P, Suter M, Hausmann J. 2007. Genomic sequence of chorioallantois vaccinia virus Ankara, the ancestor of modified vaccinia virus Ankara. J Gen Virol 88:3249–3259. doi:10.1099/vir.0.83156-0 18024893

[6] Meisinger-Henschel C, Späth M, Lukassen S, Wolferstätter M, Kachelriess H, Baur K, Dirmeier U, Wagner M, Chaplin P, Suter M, Hausmann J. 2010. Introduction of the six major genomic deletions of modified vaccinia virus Ankara (MVA) into the parental vaccinia virus is not sufficient to reproduce an MVA-like phenotype in cell culture and in mice. J Virol 84:9907–9919. doi:10.1128/JVI.00756-10 20668072 PMC2937755

[7] Meyer H, Sutter G, Mayr A. 1991. Mapping of deletions in the genome of the highly attenuated vaccinia virus MVA and their influence on virulence. J Gen Virol 72 (Pt 5):1031–1038. doi:10.1099/0022-1317-72-5-1031 2033387

[8] Orlova OV, Glazkova DV, Bogoslovskaya EV, Shipulin GA, Yudin SM. 2022. Development of modified vaccinia virus ankara-based vaccines: advantages and applications. Vaccines (Basel) 10:1516. doi:10.3390/vaccines10091516 36146594 PMC9503770

[9] Dai P, Wang W, Cao H, Avogadri F, Dai L, Drexler I, Joyce JA, Li X-D, Chen Z, Merghoub T, Shuman S, Deng L. 2014. Modified vaccinia virus Ankara triggers type I IFN production in murine conventional dendritic cells via a cGAS/STING-mediated cytosolic DNA-sensing pathway. PLoS Pathog 10:e1003989. doi:10.1371/journal.ppat.1003989 24743339 PMC3990710

[10] Keskitalo S, Haapaniemi E, Einarsdottir E, Rajamäki K, Heikkilä H, Ilander M, Pöyhönen M, Morgunova E, Hokynar K, Lagström S, Kivirikko S, Mustjoki S, Eklund K, Saarela J, Kere J, Seppänen MRJ, Ranki A, Hannula-Jouppi K, Varjosalo M. 2019. Novel TMEM173 mutation and the role of disease modifying alleles. Front Immunol 10:2770. doi:10.3389/fimmu.2019.02770 31866997 PMC6907089

[11] Liu N, Pang X, Zhang H, Ji P. 2021. The cGAS-STING pathway in bacterial infection and bacterial immunity. Front Immunol 12:814709. doi:10.3389/fimmu.2021.814709 35095914 PMC8793285

[12] Caiazza C, Brusco T, D’Alessio F, D’Agostino M, Avagliano A, Arcucci A, Ambrosino C, Fiume G, Mallardo M. 2022. The lack of STING impairs the MHC-I dependent antigen presentation and JAK/STAT signaling in murine macrophages. Int J Mol Sci 23:22. doi:10.3390/ijms232214232 PMC9697192 36430709

[13] Xu Y, Xiong Y. 2024. Targeting STING signaling for the optimal cancer immunotherapy. Front Immunol 15:1482738. doi:10.3389/fimmu.2024.1482738 39450170 PMC11500076

[14] Chen Q, Sun L, Chen ZJ. 2016. Regulation and function of the cGAS-STING pathway of cytosolic DNA sensing. Nat Immunol 17:1142–1149. doi:10.1038/ni.3558 27648547

[15] Ikeda F, Hecker CM, Rozenknop A, Nordmeier RD, Rogov V, Hofmann K, Akira S, Dötsch V, Dikic I. 2007. Involvement of the ubiquitin-like domain of TBK1/IKK-i kinases in regulation of IFN-inducible genes. EMBO J 26:3451–3462. doi:10.1038/sj.emboj.7601773 17599067 PMC1933404

[16] Motwani M, Pesiridis S, Fitzgerald KA. 2019. DNA sensing by the cGAS-STING pathway in health and disease. Nat Rev Genet 20:657–674. doi:10.1038/s41576-019-0151-1 31358977

[17] Tamura T, Yanai H, Savitsky D, Taniguchi T. 2008. The IRF family transcription factors in immunity and oncogenesis. Annu Rev Immunol 26:535–584. doi:10.1146/annurev.immunol.26.021607.090400 18303999

[18] Liu T, Zhang L, Joo D, Sun S-C. 2017. NF-κB signaling in inflammation. Signal Transduct Target Ther 2:17023. doi:10.1038/sigtrans.2017.23 29158945 PMC5661633

[19] Froechlich G, Caiazza C, Gentile C, D’Alise AM, De Lucia M, Langone F, Leoni G, Cotugno G, Scisciola V, Nicosia A, Scarselli E, Mallardo M, Sasso E, Zambrano N. 2020. Integrity of the antiviral STING-mediated DNA sensing in tumor cells is required to sustain the immunotherapeutic efficacy of herpes simplex oncolytic virus. Cancers (Basel) 12:11. doi:10.3390/cancers12113407 PMC7698602 33213060

[20] Perng YC, Lenschow DJ. 2018. ISG15 in antiviral immunity and beyond. Nat Rev Microbiol 16:423–439. doi:10.1038/s41579-018-0020-5 29769653 PMC7097117

[21] Li C, Du S, Tian M, Wang Y, Bai J, Tan P, Liu W, Yin R, Wang M, Jiang Y, Li Y, Zhu N, Zhu Y, Li T, Wu S, Jin N, He F. 2018. The host restriction factor interferon-inducible transmembrane protein 3 inhibits vaccinia virus infection. Front Immunol 9:228. doi:10.3389/fimmu.2018.00228 29503647 PMC5820317

[22] Mallardo M, Leithe E, Schleich S, Roos N, Doglio L, Krijnse Locker J. 2002. Relationship between vaccinia virus intracellular cores, early mRNAs, and DNA replication sites. J Virol 76:5167–5183. doi:10.1128/jvi.76.10.5167-5183.2002 11967332 PMC136133

[23] Guerra S, Cáceres A, Knobeloch K-P, Horak I, Esteban M. 2008. Vaccinia virus E3 protein prevents the antiviral action of ISG15. PLoS Pathog 4:e1000096. doi:10.1371/journal.ppat.1000096 18604270 PMC2434199

[24] Ishikawa H, Ma Z, Barber GN. 2009. STING regulates intracellular DNA-mediated, type I interferon-dependent innate immunity. Nature 461:788–792. doi:10.1038/nature08476 19776740 PMC4664154

[25] Diamond MS, Farzan M. 2013. The broad-spectrum antiviral functions of IFIT and IFITM proteins. Nat Rev Immunol 13:46–57. doi:10.1038/nri3344 23237964 PMC3773942

[26] Lin C, Kuffour EO, Fuchs NV, Gertzen CGW, Kaiser J, Hirschenberger M, Tang X, Xu HC, Michel O, Tao R, Haase A, Martin U, Kurz T, Drexler I, Görg B, Lang PA, Luedde T, Sparrer KMJ, Gohlke H, König R, Münk C. 2023. Regulation of STING activity in DNA sensing by ISG15 modification. Cell Rep 42:113277. doi:10.1016/j.celrep.2023.113277 37864791

[27] Chen F, Wu R, Liu J, Kang R, Li J, Tang D. 2022. The STING1-MYD88 complex drives ACOD1/IRG1 expression and function in lethal innate immunity. iScience 25:104561. doi:10.1016/j.isci.2022.104561 35769880 PMC9234224

[28] Cheng Y, Sun Y, Wang H, Yan Y, Ding C, Sun J. 2015. Chicken STING mediates activation of the IFN gene independently of the RIG-I gene. J Immunol 195:3922–3936. doi:10.4049/jimmunol.1500638 26392466

[29] Tolonen N, Doglio L, Schleich S, Krijnse Locker J. 2001. Vaccinia virus DNA replication occurs in endoplasmic reticulum-enclosed cytoplasmic mini-nuclei. Mol Biol Cell 12:2031–2046. doi:10.1091/mbc.12.7.2031 11452001 PMC55651

[30] Deng Y, Navarro-Forero S, Yang Z. 2025. Temporal expression classes and functions of vaccinia virus and mpox (monkeypox) virus genes. mBio 16:e0380924. doi:10.1128/mbio.03809-24 40111027 PMC11980589

[31] Guerra S, López-Fernández LA, Conde R, Pascual-Montano A, Harshman K, Esteban M. 2004. Microarray analysis reveals characteristic changes of host cell gene expression in response to attenuated modified vaccinia virus Ankara infection of human HeLa cells. J Virol 78:5820–5834. doi:10.1128/JVI.78.11.5820-5834.2004 15140980 PMC415835

[32] Ren H, Wang S, Xie Z, Wan L, Xie L, Luo S, Li M, Xie Z, Fan Q, Zeng T, Zhang Y, Zhang M, Huang J, Wei Y. 2024. Analysis of chicken IFITM3 gene expression and its effect on avian reovirus replication. Viruses 16:330. doi:10.3390/v16030330 38543696 PMC10974799

[33] Osei Kuffour E, König R, Häussinger D, Schulz WA, Münk C. 2019. ISG15 deficiency enhances HIV-1 infection by accumulating misfolded p53. mBio 10:e01342-19. doi:10.1128/mBio.01342-19 31455647 PMC6712392

[34] Schmitz F, Heit A, Guggemoos S, Krug A, Mages J, Schiemann M, Adler H, Drexler I, Haas T, Lang R, Wagner H. 2007. Interferon-regulatory-factor 1 controls Toll-like receptor 9-mediated IFN-beta production in myeloid dendritic cells. Eur J Immunol 37:315–327. doi:10.1002/eji.200636767 17273999

[35] Hood AJM, Sumner RP, Maluquer de Motes C. 2022. Disruption of the cGAS/STING axis does not impair sensing of MVA in BHK21 cells. J Gen Virol 103. doi:10.1099/jgv.0.001755 35584007

[36] Lin C, Zheng M, Xiao S, Wang S, Zhu X, Chen X, Jiang D, Zeng X, Chen S, Chen S. 2023. Duck cGAS inhibits DNA and RNA virus replication by activating IFNs and antiviral ISGs. Front Immunol 14:1101335. doi:10.3389/fimmu.2023.1101335 36733488 PMC9887016

[37] Motani K, Kosako H. 2020. BioID screening of biotinylation sites using the avidin-like protein Tamavidin 2-REV identifies global interactors of stimulator of interferon genes (STING). J Biol Chem 295:11174–11183. doi:10.1074/jbc.RA120.014323 32554809 PMC7415991

[38] Di Lullo G, Soprana E, Panigada M, Palini A, Erfle V, Staib C, Sutter G, Siccardi AG. 2009. Marker gene swapping facilitates recombinant modified vaccinia virus Ankara production by host-range selection. J Virol Methods 156:37–43. doi:10.1016/j.jviromet.2008.10.026 19038289

