# Supplementary material for: STING-mediated antiviral response: insights into MVA replication control in avian cells
Source: Microbiol Spectr. 2025 Sep 22;13(11):e00075-25. doi: 10.1128/spectrum.00075-25 (PMC12584662; doi:10.1128/spectrum.00075-25)
Supplement: Supplemental material — Fig. S1 and S2; Table S1. [file spectrum.00075-25-s0001.docx]

**SUPPLEMENTARY FIGURES**

**
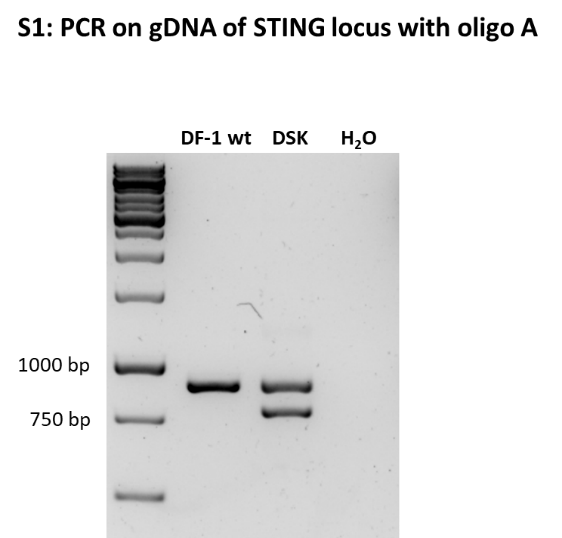
**

Figure S1. PCR on gDNA of STING locus with oligo A.

Genomic DNA screening analysis by PCR of the STING locus in DF-1 and DSK. H_2_O is used as negative control in the PCR reaction.

**
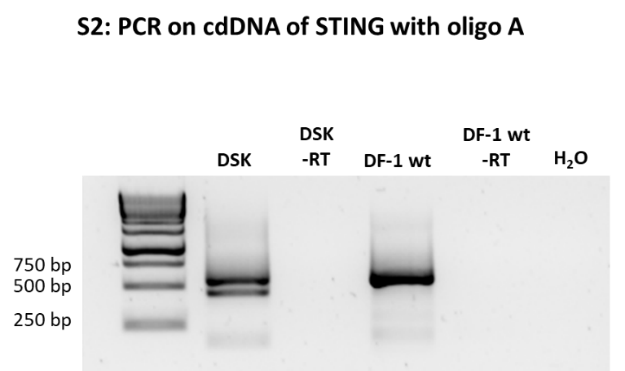
**

Figure S2. PCR on cDNA of STING with oligo A.

STING expression analyzed by PCR on cDNA of DF-1 and DSK. RT samples for each cell lines and H_2_O are used as negative control in the PCR reaction.

**Supplementary Material**

**Table S1.** Oligonucleotides sequence used for RT-PCR analysis.

| **Target** | **Forward (5’-3’)** | **Reverse (5’-3’)** |
| --- | --- | --- |
| STING “Oligo B” | TGCTCGCCTTCTCATCCCTG | CAGGAACAGCACCACGAAGC |
| STING “Oligo C” | CAGGTGCTGTGTTCCTGCTTC | GGCTGTGCCCATTGTCCTC |
| GAPDH [39] | AGGACCAGGTTGTCTCCTGT | CCATCAAGTCCACAACACGG |
| INFα | ATGCCACCTTCTCTCACGAC | AGGCGCTGTAATCGTTGTCT |
| INFβ [39] | CCTCAACCAGATCCAGCATTAC | CCCAGGTACAAGCACTGTAGTT |
| F1L | TCTGGTGGTGAAATGTCCGT | GGTTGGGTGTAAGATTGGCG |
| K3L | TCTGCCAAGATAGCTTCAGAGT | GCGGGCGATGTAATAAAGGG |
| E3L | AGGAATATCGTCGGAGCTGT | GAGCGTTCTGACGCAGAGAT |
| B16R | ACGCGCCTGAATGTATCGAC | ATCCGCTCCTCGTTTTTCCC |
| D8 | TGGGGAGAAACCCTCCACTT | GCGCGATTGAAGCCGTTAG |
| ISG15 | TGCCAGTACAGGAGCTTGTG | GTCAGGTCCCAGCCCATG |
| Myd88 [40] | TGATGCCTTCATCTGCTACTG | TCCCTCCGACACCTTCTTTCTA |
| IFITM3 [41] | GGAGTCCCACCGTATGAAC | GGCGTCTCCACCGTCACCA |
| IL-8 [42] | TTGGAAGCCACTTCAGTCAGAC | GGAGCAGGAGGAATTACCAGTT |
| IRF1 [43] | GCTACACCGCTCACGA | TCAGCCATGGCGATTT |
